# Why we have to move beyond the idea of cultural competency

**DOI:** 10.1080/10872981.2020.1841398

**Published:** 2020-10-28

**Authors:** Sarah Wong, Christina Plowman, Davina Puri, Ife Nwibe

**Affiliations:** Medical School, University College London, London, UK

Dear Editor,

We are writing this letter in response to the article by Baugh, Vanderbilt and Baugh [1] regarding cultural proficiency training in medical schools and the bearing of cultural factors on clinical communication. As a group of senior medical students involved in a movement to decolonize – that is, to recognize how historical forces of racism and empire continue to shape power hierarchies present within [2] – the medical curriculum, we recognize that this encompasses dismantling the power imbalances within the clinician-patient encounter. Our backgrounds in medical anthropology have made us cognizant of examples in clinical practice where individual patients have been disadvantaged or disempowered by a clinician’s biased attitudes towards them, whether these were conveyed in explicit or implicit ways. We share the authors’ concerns about how this affects trust in the clinician-patient relationship and long-term health outcomes and agree with their problematization of the skill-performance mindset that may arise from the cultural competency/proficiency model. However, we contend that the problem lies in the conceptual basis of this model rather than in ‘limited experiential exposure to different cultures’ [1]. The core premise of cultural ‘competency’ assumes that it is possible to acquire the skill to navigate cross-cultural barriers in clinical settings through sufficient knowledge, training and practice. As Kumagai and Lypson suggest, ‘cultural competency is not an abdominal exam’ [3]. Yet, this is how it is so often framed within the medical curriculum: as a clinical skill to be checked off a list. From our experience, cultural competence training is often confined to an isolated session that explores fictitious clinical scenarios inspired by ethnic minority stereotypes, and students are taught what constitutes an ‘appropriate’ response within each encounter. Unlike the physical abdominal exam, which can be learnt and performed start-to-finish in a methodical manner, navigating culture is a much more abstract, complex and intricate endeavor. Thus, responding to cultural differences must be a continuous, reflexive process throughout the clinical encounter.

Recently our medical school invited us to organize a session on ‘cultural competency’ for incoming medical students, which we reframed as ‘cultural safety’ in an effort to shift the paradigm around the discussion of what constitutes culture. Cultural safety is a model for healthcare training that emphasizes a continuous process of self-reflection and engagement with the perspectives of others (i.e. patients) that produces awareness of one’s own (i.e. the clinician’s) biases, dispositions and assumptions [4]. Furthermore, this model foregrounds how institutional racism and other systemic barriers to healthcare access contribute to a disproportionate health burden on ethnic minority and indigenous populations. In our teaching session, we examined the concept of culture through an intersectional lens, critiquing the use of ‘culture’ as a proxy term for race or ethnicity and instead presenting it as a much more dynamic construct – a set of values, beliefs and behaviors embodied by a collective group. We asked students to examine the multiple cultures they belong to and shift between, as well as envision what a culturally safe environment within medical school and in online settings would look like for themselves and their peers. The feedback we received from the session was positive, and students even suggested extending the time for the session and having more opportunities for interaction, sharing and reflection.

The ability to respond to cultural difference in a way that does not homogenize, exoticize or marginalize the individual in focus involves a level of reflexivity that the cultural competency model, in emphasizing the otherness of the patient relative to the doctor, simply does not account for. Baugh et al. recognize that effective communication skills cannot be taught in an artificial setting and suggest cultural exposure programs as a means to foster empathy. However, experience alone does not equate empathy, and empathy alone is not enough to dismantle the power structures that hinder doctor-patient communication and the realization of an equitable health system. Instead, cross-cultural communication skills must be cultivated throughout the course of medical school, integrated into all aspects of the curriculum to help students nurture and preserve a disposition towards cultural humility. The type of exposure we really need in medical school is that which teaches us the limits of our own knowledge. This includes learning from and valuing alternative perspectives, ideas and ways of thinking in relation to ourselves so that we may become culturally safe clinicians.
